# Cervical Malignant Melanotic Nerve Sheath Tumor with retained PRKAR1A expression and a clinically benign course: a case report and review of the literature

**DOI:** 10.3389/fsurg.2025.1618362

**Published:** 2025-09-15

**Authors:** Alexandru Guranda, Johannes Wach, Erdem Güresir, Max Braune, Peter Kuzman, Ulf Nestler

**Affiliations:** ^1^ Department of Neurosurgery, University Hospital Leipzig, Leipzig, Germany; ^2^ Paul-Flechsig-Institute of Neuropathology, University Hospital Leipzig, Leipzig, Germany

**Keywords:** malignant melanotic nerve sheath tumor (MMNST), cervical nerve root, melanotic schwannoma, psammomatous melanotic schwannoma, case report

## Abstract

**Background:**

Malignant melanotic nerve sheath tumors (MMNSTs) are rare Schwann cell–derived tumors. Previously classified as benign melanotic schwannomas, they were redefined as a potentially aggressive entity in the 2020 WHO classification of soft tissue tumors and later included in the 2021 WHO CNS classification. However, optimal therapeutic strategies remain under discussion.

**Case presentation:**

We present the case of a 46-year-old Caucasian male who underwent surgery for an intra- and extraspinal cervical mass lesion at the C4/5 level on the left side. Immunohistochemical analysis confirmed the diagnosis of MMNSTs. The patient initially presented with ataxia, left-sided weakness, and hemihypesthesia. Magnetic resonance imaging of the cervical spine revealed a left intra- and extraspinal homogeneous contrast-enhancing mass at the C4/C5 level. After the first intraspinal partial resection, the diagnosis of MMNST was established. Gross-total resection is highly recommended in nearly all cases in the literature, followed by adjuvant radiotherapy or chemotherapy in selected cases to prevent metastases, which occur in 15%–42% of cases. The patient postponed the second neurosurgical intervention and declined adjuvant radiotherapy. At 18 months after gross total resection, no recurrent tumor was detected by MRI.

**Conclusion:**

Given the limited epidemiological knowledge on MMNSTs, our study contributes to the literature by documenting a case of intra- and extraspinal, cervical MMNST without any of the previously known driver mutations or copy number changes. While the WHO 2021 classification designates these tumors as potentially malignant, our findings support existing reports that more benign courses can occur.

## 1 Introduction

Malignant melanotic nerve sheath tumor (MMNST) is a rare peripheral nerve sheath tumor showing both Schwann cell and melanocytic differentiation. Although melanotic schwannomas have historically been considered as rather benign lesions, the 2021 WHO classification of central nervous system tumors defines MMNSTs as a potentially aggressive entity (1, 2).

MMNSTs often harbor PRKAR1A mutations, particularly in patients with Carney complex (3), an autosomal dominant syndrome associated with cardiac myxomas (4), pigmented skin lesions, and endocrine tumors (5). Loss of PRKAR1A expression has been proposed as a key molecular event in MMNST pathogenesis, leading to dysregulated cAMP signaling and tumorigenesis (3). However, not all MMNST cases exhibit PRKAR1A loss, indicating potential alternative oncogenic mechanisms, such as aberrations in the MAPK or mTOR pathways (6). Sporadic MMNSTs frequently retain PRKAR1A expression (7).

Despite their classification as malignant, MMNSTs exhibit variable clinical behavior, ranging from indolent locally controlled disease to aggressive tumors with distant spread. Metastases have been reported, most commonly in the lungs (8), liver (9), and bones (10). Due to this variability, there is no standardized treatment protocol, and management remains largely individualized. While gross total resection (GTR) is the preferred treatment (11, 12), the role of adjuvant radiotherapy and systemic therapy remains uncertain, particularly in cases with complete surgical removal and no high-risk histopathological features. Some reports suggest that radiotherapy may improve local control (13, 14), while others argue that it can be postponed in cases without aggressive histopathology (15).

Our case presents a cervical intra- and extraspinal MMNST without PRKAR1A mutation or copy number changes, a finding rarely documented in the literature. Since the most recent WHO classification, 18 intraspinal MMNSTs have been reported, including four located in the cervical spine and one at the cervicothoracic junction. This rare occurrence, with its unique molecular profile and favorable clinical course, contributes to the ongoing discussion of which MMNST subtypes exhibit aggressive behavior and which cases may warrant adjuvant therapy.

## 2 Case presentation

**Figure 1:**
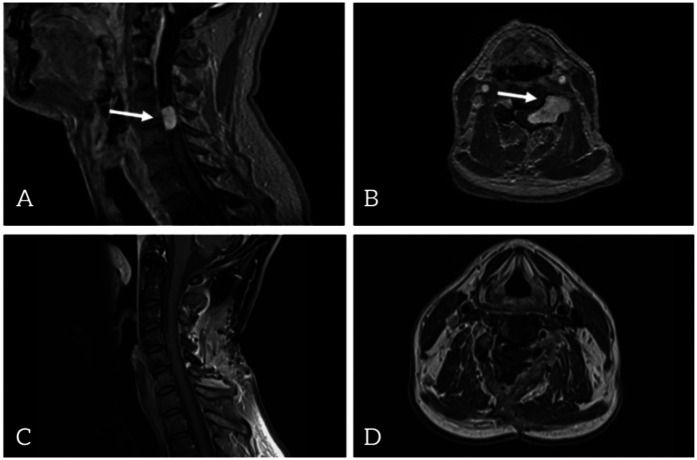
Contrast-enhanced T1-weighted magnetic resonance imaging (MRI) preoperative images: sagittal view **(A)** and axial view **(B)** postoperative images: sagittal view **(C)** and axial view **(D)**.

A 46-year-old Caucasian male carpenter with a 6-week history of left-sided weakness and hemihypesthesia was admitted to the Department of Neurosurgery. Initial cranial imaging ruled out multiple sclerosis and stroke. Subsequent imaging revealed a contrast-enhancing intraspinal mass at the level of C4/C5, extending to the left side and encasing the left C5 nerve root up to the vertebral artery (Figure 1).

Clinical examination revealed adduction paresis of the fingers of the left hand (muscle strength grade 3/5), atrophy of the interosseous muscles, distally accentuated hypesthesia of the left upper extremity, spasticity of the left lower extremity, and an unsteady gait with a tendency to fall to the left. Routine laboratory parameters, including markers of infection and inflammation, were within normal limits.

The patient was very anxious and initially reluctant to undergo the proposed treatment. Only six weeks after the initial diagnosis he agreed to neurosurgical resection. Intraoperatively, the tumor was encapsulated by white, fibrous tissue, compressing the spinal cord and displacing it to the midline. Inside the capsule, black granular tissue triggered suspicion of melanoma, which was supported by the intraoperative frozen section analysis. Significant bleeding complicated the initial surgery. Due to persistent hemorrhage and the suspicion of malignancy, surgical resection was halted after intradural decompression of the spinal cord and partial lateral tumor removal.

Following surgery, the patient initially presented with persistent hypesthesia of the right arm, distal weakness of the left arm with a muscle strength grade of 4/5, and proximal weakness of the right arm with a strength grade of 3/5. Over the course of follow-up, a gradual improvement was observed. The abduction weakness in the right arm persisted with a strength grade of 3/5, while left arm flexion improved to 4/5. Hypesthesia in the right arm showed regression. Additionally, gait steadiness improved progressively.

**Figure 2:**
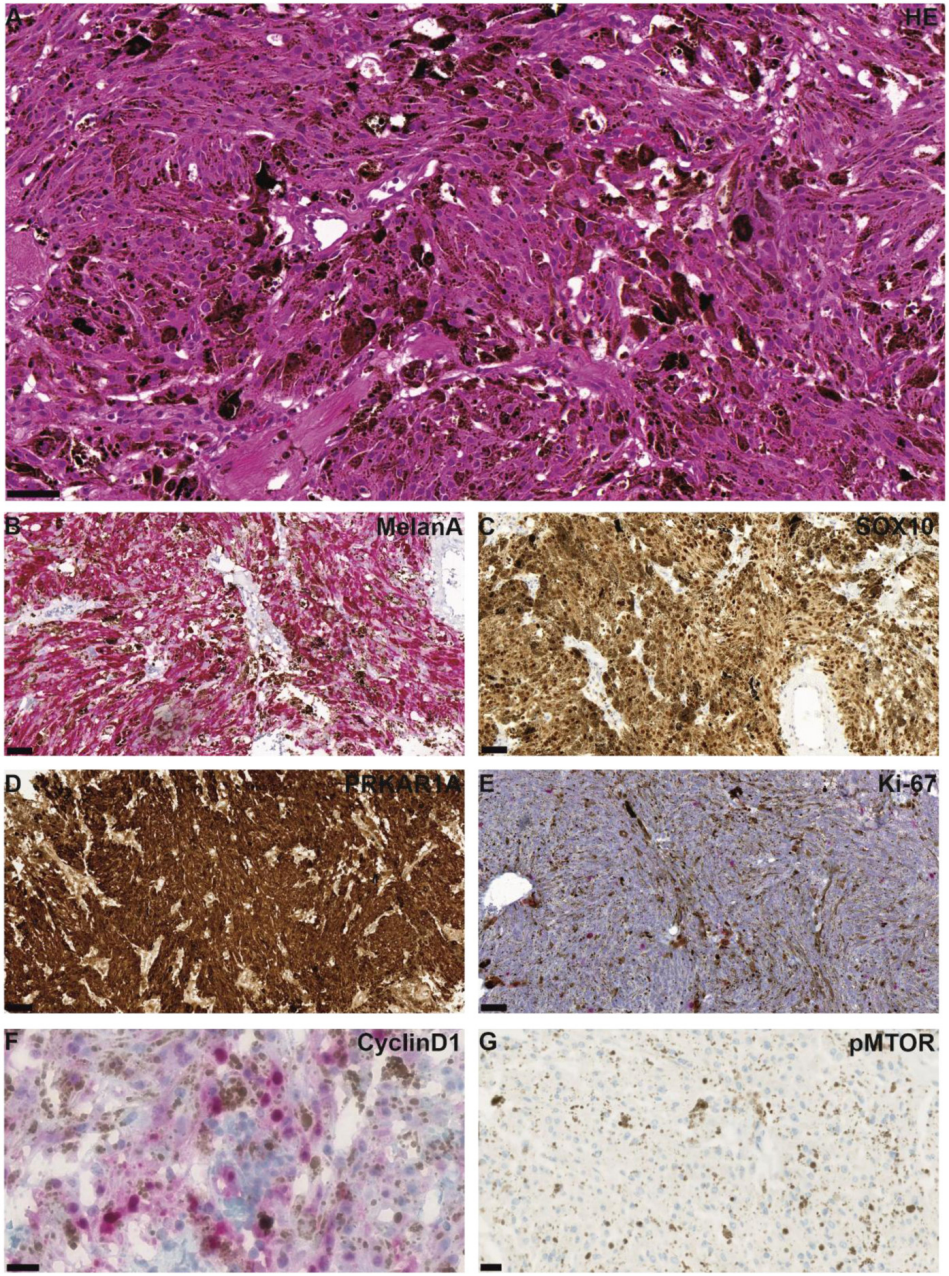
Representative microphotographs of HE and immunohistochemical staining are shown. HE staining shows a tumor with epithelioid to spindle-shaped morphology and heavy melanotic pigmentation, arranged in short fascicles **(A)** Tumor cells were positive for MelanA **(B)** and SOX10 **(C)** PRKAR1A was retained **(D)** Proliferation was low, as indicated by Ki67 staining **(E)** Immunohistochemical staining for Cyclin D1 showed partial nuclear positivity in tumor cells **(F)**, whereas no reactivity was observed for mTOR **(G)** Scale bars are 50 µm **(A–C,E)**, 100 µm **(D)**, and 20 µm **(F,G)**.

Histologically, the tumor showed an epithelioid to spindle-shaped morphology with heavy melanotic pigmentation and moderate cellularity, arranged in short fascicles (Figure 2A). There were no signs of cellular atypia, mitoses or necrosis. There were no psammoma bodies.

The tumor tissue demonstrated broad immunohistochemical positivity for S-100, MelanA, and HMB45, as well as for Sox10 (Figures 2B,C). In the current case, PRKAR1A immunoexpression was retained (Figure 2D). Ki67 immunostaining showed a low proliferative index of approximately 3% (Figure 2E).

Furthermore, molecular mutation detection and gene fusion analysis were initiated to exclude other morphological mimics of melanocytic tumors (primary CNS melanocytoma and melanoma, metastatic cutaneous melanoma). While these targeted analyses provided relevant molecular information, whole-exome sequencing (WES) was unfortunately not available due to technical reasons.

DNA methylation profiling was performed on formalin-fixed, paraffin-embedded (FFPE) tissue from both the primary and recurrent tumor using the Illumina Infinium MethylationEPIC BeadChip array (850k). Raw data were normalized and quality-controlled using the ChAMP R package. 17849 differentially methylated positions (DMPs) between the tumor samples and conventional schwannoma controls (*n* = 20) were identified using the champ.DMP() function, which is based on the *limma* package to fit linear models across all CpG sites. Analyses were conducted on M-values (log₂-transformed beta values) The Benjamini–Hochberg procedure was applied to control the false discovery rate (FDR). DMPs with an adjusted *p*-value < 0.05 were considered statistically significant. Gene sets related to the MAPK and mTOR signaling pathways were retrieved from the Molecular Signatures Database (MSigDB) via the *msigdbr* R package. DMPs associated with MAPK and mTOR signaling pathways were extracted for Gene set enrichment analysis (GSEA) using the *clusterProfiler* package, based on log-fold changes aggregated at the gene level from DMP results.

**Figure 3:**
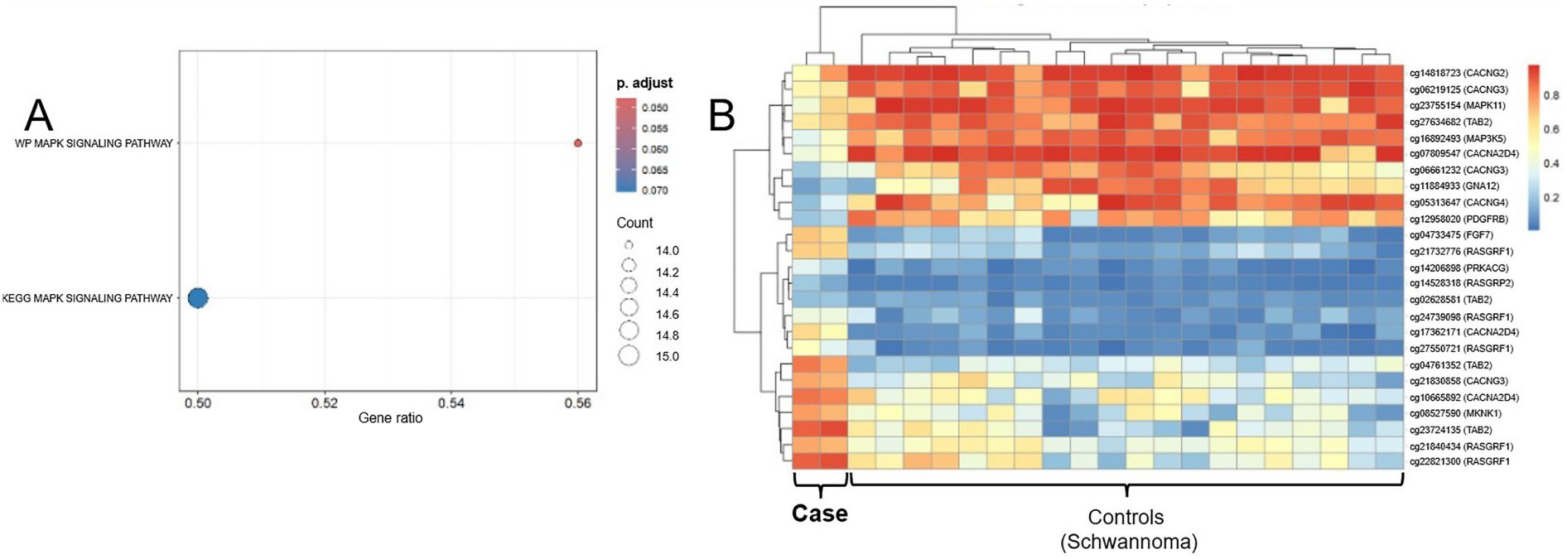
**(A)** Dot plot of gene set enrichment analysis (GSEA) highlighting KEGG and wikiPathways terms related to MAPK signaling. The *x*-axis represents the gene ratio (proportion of input genes contributing to each pathway), while dot size reflects the number of core genes (*gene count*) involved in each term. Color intensity indicates the statistical significance (adjusted *p*-value) of enrichment. *WP_MAPK_SIGNALING_PATHWAY* shows significant enrichment (*adjusted p* = 0.046), whereas *KEGG_MAPK_SIGNALING_PATHWAY* approaches significance (*adjusted p* = 0.070), supporting MAPK pathway involvement in the tumor. **(B)** Heatmap of methylation β-values for CpG sites associated with MAPK core genes. CpG loci (rows) are annotated with the corresponding gene symbols [e.g., “cg12345678 (*MAP3K5*)”]. Columns represent individual tumor and schwannoma control samples. Methylation levels are color-coded from hypomethylated (blue) to hypermethylated (red). Hierarchical clustering reveals a distinct methylation pattern of MAPK-related CpGs in the tumor, supporting epigenetic involvement of the MAPK signaling pathway.

Significant enrichment of genes involved in the MAPK signaling pathway was observed (e.g., MAP3K5, MAPK11, PDGFRB, RASGRF1). In contrast, no significant enrichment of genes involved in the mTOR signaling pathway was detected (Figure 3A). A heatmap of associated CpG sites using beta values revealed distinct methylation patterns compared to controls (Figure 3B). Immunohistochemical staining demonstrated partial Cyclin D1 expression, supporting functional activation of the MAPK pathway, whereas no mTOR staining was observed (Figures 2F,G), consistent with the epigenetic findings. It should be noted that heavy melanin pigmentation limited the interpretation of the staining. Therefore, based on these results alone, mTOR expression cannot be definitively excluded, and precise quantification of Cyclin D1-positive cells remains challenging.

Targeted next-generation sequencing (NGS) using a customized hybrid-capture panel covering all coding regions as well as selected intronic and promoter regions of 130 genes [as described by Sahm et al. (16)] revealed no mutations in PRKAR1A, BRAF, NRAS, GNAQ, GNA11, KIT, TERT, or any other gene included in the panel.

**Figure 4:**
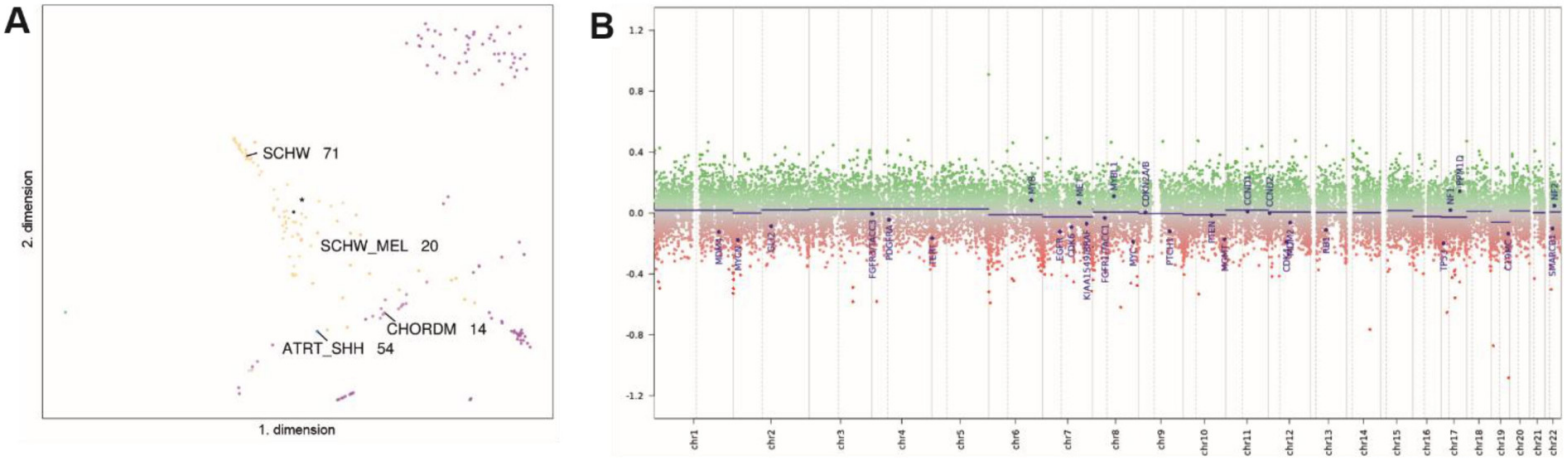
tSNE analysis shows clustering with MMNSTs (black dot, marked with asterisk, SCHW_MEL). Proximal methylation classes include Schwannomas (SCHW), atypical teratoid/rhabdoid tumors, subgroup SHH (ATRT_SHH), and chordoma (CHORDM) **(A)** The copy number profile is flat, showing no chromosomal gains or losses. The copy number calculation was obtained from the publicly available database of the German Cancer Research Center (DKFZ) at www.molecularneuropathology.org. and is based on the conumee package **(B)**.

Mutation detection and gene fusion analysis were performed using the customized panels QIASeq targeted DNA Panel for Solid Tumors (Qiagen) and QIAseq targeted RNAscan Panel (Qiagen). High-throughput sequencing was then performed on a MiSeq (Illumina) instrument, which revealed wild-type sequences for the BRAF, NRAS, KIT, TERT, GNAQ, and GNA11 genes. Additionally, genome-wide DNA methylation analysis was performed using the EPIC Illumina Human Methylation 850 (850k) array v1.0. The methylation profile of the tumor was compared with previously defined methylation classes using the publicly available database of the German Center for Cancer Research (DKFZ) (17) via www.molecularneuropathology.org. The brain tumor classifiers v11b.4 and v12.5 showed the highest score for the methylation class of malignant melanotic nerve sheath tumor in the primary tumor (score: 0.71) and the extraspinal tumor remnant (score: 0,99), which could be confirmed via unsupervised tSNE analysis using the DistSNE platform (Figure 4A) (18). Copy number profiling revealed none of the changes reported in the literature (Figure 4B) (19), and no copy number alteration was observed at the PRKAR1A locus.

Interestingly, the sarcoma classifier showed a match in the primary tumor for soft tissue clear cell sarcoma (Score: 0.96), but EWSR1/CREB1 or ATF1 gene fusion were excluded. Additionally, mutation analysis of PRKAR1A was performed using a customized enrichment/hybrid-capture-based panel of genes recurrently altered in brain tumors (16). This analysis revealed no PRKAR1A mutation, thereby confirming the immunohistochemical result. For histopathological examination, the FFPE tissue was sent to the German reference center for soft tissue and bone tumors, where the diagnosis was confirmed.

This case represents an interesting molecular profile of an MMNST without PRKAR1A mutation, without any of the reported copy number changes, and without other previously known driver mutations.

## 3 Postoperative course

Complete resection followed by radiotherapy was recommended by the sarcoma board and the interdisciplinary neuro-oncology board. At the time of considering a second neurosurgical intervention, the patient had not yet been able to resume his profession as a painter. Although gait and postural stability had significantly improved after the initial surgery, he continued to experience sensory disturbances in the left shoulder radiating to both thumbs and index fingers, more pronounced on the left side. Additionally, he reported paresthesia predominantly affecting the right thumb and persistent neck pain exacerbated by forward bending. Although no paresis of the upper extremities was present, he still suffered from exertional weakness. Only after careful consideration of the persistent symptoms and the recommendations did the patient agree to a second neurosurgical intervention, which was performed five months after the initial partial resection.

This time, according to the view of the operative microscope and confirmed by early postoperative MRI (Figure 1), complete resection was achieved. The paresis further improved, and the patient reported no complaints. During follow-up, the patient described paresthesia on the left side and noted similar sensations extending to his right thumb. His gait became steady.

Upon clinical reassessment based on the histologic diagnosis, the patient exhibited no clinical features indicative of Carney complex, such as pigmented skin lesions, cardiac myxoma, or endocrine dysfunction. Additionally, there was no family history of Carney complex or related syndromes.

Subsequently, the patient agreed to undergo oncologic and radiotherapeutic counseling but continued to decline radiotherapy. His decision was influenced by the significant improvement in symptoms and the absence of recurrence on MRI. The most recent MRI, performed 18 months after the initial surgery, showed no evidence of recurrent disease. At the two-year follow-up, the patient reported only mild, persistent paresthesia.

## 4 Discussion

We describe a rare case of a cervical MMNST with intra- and extraspinal extension, absence of PRKAR1A mutation, absence of detectable copy number changes, and a benign clinical course without adjuvant therapy. The patient remained recurrence-free for 24 months after gross total resection, suggesting that not all MMNSTs exhibit aggressive behavior. These findings are in line with recent reports of sporadic MMNSTs retaining PRKAR1A expression and lacking metastatic potential.

Malignant melanotic nerve sheath tumors account for less than 1% of all primary peripheral nerve sheath tumors and most often arise from the spinal nerves or visceral autonomic nerves (20–22). There have been documented cases in various anatomical locations, including the stomach, bone, soft tissue, heart, liver, choroid, and skin (23, 24).

MMNST has historically been regarded as an atypical variant of schwannoma and, due to its pigmentation, has been referred to as melanotic schwannoma (25). The first documented case dates back to 1932, and in 1975 the close relationship of these tumors to the sympathetic chain, as well as their metastatic potential, was described (25–27).

In some series, over 50% of patients with MMNSTs have evidence of Carney complex, an autosomal dominant, occasionally familial multiple neoplasia syndrome (27). However, other studies have reported an association with Carney complex in less than 5% of affected patients (19, 28–31). Some cases occur sporadically with an unknown etiology (27). MMNSTs associated with Carney complex more frequently display psammomatous morphology (32). A few reported cases have shown an association with neurofibromatosis (33). Importantly, the patient in our study did not exhibit any clinical features of Carney complex or neurofibromatosis. Psammomatous MMNSTs have more often been reported in association with Carney complex (29, 34), and PRKAR1A alterations are common in melanotic schwannoma (19). Nevertheless, both psammomatous and non-psammomatous cases may variably harbor these alterations, and clinical behavior remains heterogeneous (3). Our case, which displayed neither psammomatous features nor PRKAR1A loss, aligns with these latter reports. In such cases, alternative oncogenic pathways such as mTOR and MAPK activation must be considered. These findings underscore the heterogeneity of MMNSTs and emphasize the need for detailed molecular studies to define treatment strategies more precisely.

The average age at diagnosis of MMNST is 41 years, with a reported range of 11–81 years, which corresponds well to our patient (46 years) (29). However, some sources suggest a mean age of 38 years (29, 30). It is important to note that there is no gender or ethnic predilection (20, 34).

Table 1 summarizes cases identified in PubMed using the search terms ‘‘MMNST’’ and ‘‘malignant melanotic nerve sheath tumor’’ that were published after the 2021 WHO CNS tumor classification, in which MMNST was designated a potentially aggressive condition. According to the cases summarized in Table 1, the average patient age is 42.9 years (range, 7–79 years). Sixteen patients were female and twelve male. Eighteen tumors were located in the spine, including four in the cervical region and one at the cervicothoracic junction.

**Table 1:** Recently published MMNST cases since the 2021 WHO CNS classification.

Authors	Intraspinal	Extraspinal	Year	Sex	Age (years)	Radiotherapy	PFS (months)	Follow-up (months)
Yeom et al. (44)	T11–T12		2022	F	58	No	n.a.	n.a.
	T11		2022	M	72	No (biopsy only)	36^a^	36
Terry et al. (3)	S2		2022	F	48	Yes (59.4 Gy/33 fr)	5	18
Hall et al. (45)	S1		2022	F	18	Yes (40 Gy/f fr)	30^a^	30
Yan et al. (46)	S2		2022	M	33	Yes (56 Gy)	15^a^	15
Buckley et al. (47)		Pararenal	2022	M	70	No	n.a.	n.a.
Zlatarov et al. (33)		Adrenal gland	2022	F	11	No	2^a^	2
Lin et al. (48)		Para-aortic area	2022	F	59	Yes	11^a^	11
Jackson et al. (49)		Presacral and Pleural effusion	2022	F	60	Yes	n.a.	8
Grandmougin et al. (50)	C2–C3		2023	F	31	No	n.a.	n.a.
Shui et al. (51)	L5–S1		2022	F	21	Yes (after leptomeningeal spread)	4	n.a.
Li et al. (52)		Parotid gland	2023	M	52	No	n.a.	n.a.
Bonomo et al. (21)	C5–C6		2023	F	28	No	12	12
Rachao et al. (53)		Cavernous sinus	2023	M	42	Yes (54 Gy/30fr)	n.a.	n.a.
Xiang et al. (54)	T6–T7		2023	M	60	No	n.a.	n.a.
McCann et al. (55)	T8–T11		2023	M	40	Planned	3^a^	3
Chen P et al. (31)		Retroperitoneal	2024	F	47	No	36	36
Kageyama (56)	T9–T10		2024	M	79	No	12	12
Chen S et al. (57)	C7–T1		2024	F	27	No	7	n.a.
Agostini et al. (58)		Meckel's cave	2024	F	31	Planned	n.a.	n.a.
Franca et al. (59)	C4–C5		2024	F	50	Yes	1	12
Kallen et al. (60)		Retroperitoneal	2024	F	38	No	9	13
Okal et al. (61)	L5–S2		2024	M	7	Yes (31fr)	12^a^	12
Chong et al. (62)	S1–S3		2024	M	46	No	1	5
Sun et al. (63)	L5–S1		2024	F	55	Yes	12^a^	12
Madesh et al. (64)		Posterior fossa	2025	F	42	No	n.a.	n.a.
Haq et al. (65)	L5–S2		2025	M	30	No	12^a^	12
*Present case*	*C4*–*C5*		*2025*	*M*	*46*	*No*	*24*	*24*
Summary (*n* = 28)	*n* = 18	*n* = 10		16F/12M	Mean 42.9	10 Yeas/16 No /2 Planned	Mean 12.8^b^	Mean 15.2^b^

PFS (progression-free survival), n.a. (not available), fr (fractions).

^a^ No progression at last follow-up (censored PFS).

^b^ PFS (*n* = 19) and follow-up (*n* = 18) were calculated only from cases with available data.

Radiologically, intramedullary melanotic neoplasms exhibit hyperintensity on T1-weighted MRI and hypointensity on T2-weighted MRI due to the paramagnetic effect of melanin. Extramedullary neoplasms are often confused with meningiomas, dermoids, or schwannomas (35). FDG PET/CT can help in distinguishing between benign and malignant lesions and in assessing treatment response in MMNSTs (36).

Follow-up information in the available literature is heterogeneous and often limited. Reviewing publications that also included cases reported before the 2021 WHO classification revealed equivocal treatment results. Torres-Mora et al. reported a mean follow-up of 56 months (range, 1–300 months) in 26 patients, with frequent recurrences and metastases (29). Khoo et al. provided only short-term follow-up of up to 12 months in four patients (37). Kwok et al. described 12 patients with a mean follow-up of 4.8 years (range, 1.3–10.2 years), of whom half remained disease-free, while others developed recurrence or died of disease (38). The most recent large series by Ghaith et al. summarized 71 patients and found recurrence, metastasis, and mortality rates of 42%, 27%, and 26%, respectively (39). Collectively, these data illustrate that both early and late events occur, underscoring the necessity of long-term surveillance in MMNST. To date, apart from increased mitotic activity, no specific morphological features that reliably predict malignant vs. benign behavior have been identified.

Given the scarcity of reported cases, there are currently no established guidelines for the management of this tumor. Nevertheless, gross-total resection is recommended to minimize the risk of recurrence (40–42). Additionally, it is advisable to monitor patients in the outpatient clinic, including regular lung assessments, as the lungs are the most common site of metastasis (42, 43). Radiotherapy following subtotal or gross-total resection is strongly recommended (41).

However, our patient continues to decline radiation therapy. His case underscores the heterogeneity of MMNSTs, as some tumors may follow a more indolent course. Given the absence of recurrence up to now, 24 months postoperatively, it remains uncertain in which cases immediate adjuvant therapy is needed. Regular long-term follow-up is crucial to confirm the durability of this favorable outcome.

## 5 Conclusion

Given the limited epidemiological knowledge about malignant melanotic nerve sheath tumors (MMNSTs), our study contributes to the literature by documenting a cervical intra- and extraspinal MMNST without previously described driver mutations or copy number changes. While the WHO 2021 classification designates these tumors as potentially malignant, our findings support existing reports that some cases may follow a more benign course. This case illustrates that predicting biological behavior can be challenging, even in the presence of a defined WHO classification. It is encouraging that the patient has experienced a favorable follow-up period after resection without irradiation or chemotherapy.

## Data Availability

The original contributions presented in the study are included in the article, further inquiries can be directed to the corresponding author.
